# ThRSDB: a database of Thai rice starch composition, molecular structure and functionality

**DOI:** 10.1093/database/baaa068

**Published:** 2020-12-01

**Authors:** Kwanjeera Wanichthanarak, Maysaya Thitisaksakul

**Affiliations:** Siriraj Metabolomics and Phenomics Center, Faculty of Medicine Siriraj Hospital, Mahidol University, 2 Wanglang Road, Bangkok Noi, Bangkok 10700, Thailand; Department of Biochemistry, Faculty of Science, Khon Kaen University, 123 Moo 16 Mittraphap Road, Nai-Muang, Muang District, Khon Kaen 40002, Thailand

## Abstract

As starch properties can affect end product quality in many ways, rice starch from Thai domesticated cultivars and landraces has been the focus of increasing research interest. Increasing knowledge in this area creates a high demand from the research community for better organized information. The Thai Rice Starch Database (ThRSDB) is an online database containing data extensively curated from original research articles on Thai rice starch composition, molecular structure and functionality. The key aim of the ThRSDB is to facilitate accessibility to dispersed rice starch information for, but not limited to, both research and industrial users. Currently, 373 samples from 191 different Thai rice cultivars have been collected from 39 published articles. The ThRSDB includes the search functions necessary for accessing data together with a user-friendly web interface and interactive visualization tools. We have also demonstrated how the collected data can be efficiently used to observe the relationships between starch parameters and rice cultivars through correlation analysis and Partial Least Squares Discriminant Analysis.

**Database URL**: http://thairicestarch.kku.ac.th

## Introduction

Thai rice is an important agricultural product: in 2019, the total amount of Thai rice exported was 7 580 505 metric tons, contributing $4.2 billion to the country’s GDP (1). Rice kernel quality is intrinsically determined by its starch composition, molecular structure and functionality (2). Rice starch can also be used as a biopolymer in the food, beverage and pharmaceutical industries. However, the properties of starch can greatly affect the final product quality (2, 3): the amylose to amylopectin ratio, the proportion of amylose-lipid complexes, the amylopectin branch chain length distribution, glucan chain helical conformation and granule size distribution and morphology collectively determine the functionalities of starch by modulating gelatinization, viscosity, swelling power and retrogradation (4, 5). For example, a moist and soft texture is associated with low amylose rice flour, whereas hard and porous products are caused by a large particle size of rice flour (2). Differences in the rice genotype and place of cultivation can also influence the starch characteristics, thus determining its end-uses (6–8). Therefore, rice starch properties have been extensively studied in both the academic and industrial sectors.

The properties of rice starch from Thai domesticated cultivars and landraces have been increasingly investigated leading to the greater discovery of knowledge and data generation (3, 6, 9–15). These data are vital for the research and development of rice starch-based food products such as beverages, meat products, confectionery and gluten-free products (16). However, unlike the molecular genomics for rice, where databases have become well developed and used (17–24), no database yet exists for rice starch properties. Most information is scattered in original articles and not yet organized, so extensive literature mining is needed.

The present study has established the Thai Rice Starch Database (ThRSDB) aiming to serve the rice starch research community with a digitalized, well-organized and searchable version of important Thai rice starch data. The database is an open resource freely accessible through a web interface with several functions. Various analyses were performed to examine the relationships between starch variables among Thai rice cultivars addressing the potential application of the ThRSDB.

## Materials and methods

### Data curation

The focus of ThRSDB is to collect the starch properties of Thai rice cultivars (indica subspecies) that have been reported in original research articles. The japonica rice and long-grain basmati rice grown in Thailand have not been included in the current database because they are not native Thai rice cultivars or landraces. We searched for publications indexed in the ISI, Scopus and Thai-Journal Citation Index Centre (TCI) databases using keywords such as Thai rice, Thai rice starch, Jasmine rice and cultivar names (e.g. KDML105, RD6, etc.). There were 39 published articles in both English and Thai from the literature search. The data was manually curated to obtain the observed values of key starch properties including gel consistency, granule size, amylopectin chain length distribution, gelatinization, retrogradation, viscosity, hydrolysis, moisture content, percentage amylose, percent crystallinity, swelling power and percent solubility. The amylose:amylopectin ratio was calculated as the percentage amylose divided by (100—the percentage amylose) (25). From each of the publications, both the control and treated samples were included with the treatment conditions carefully specified. A comprehensive and substantial search ensured that the cultivar names were used consistently.

### Database and web implementation

Thai Rice Starch Database (ThRSDB) is a relational database using MySQL to store and manage data. PHP 7.2 was used for data queries. The web interface was implemented using standard HTML, JavaScript and CSS running on the Apache web server. JavaScript libraries: D3.js (https://d3js.org/) and DataTables (https://datatables.net/) were for rendering interactive graphs and tables, respectively.

### Statistical analyses

The overall characteristics of the curated data such as the average values, median, standard errors (SEs) and ranges were summarized using the R package ‘psych’ (https://CRAN.R-project.org/package=psych). We demonstrated the usefulness of the curated data for rice starch research through two analyses: (i) we examined the associations between the starch parameters by calculating Pearson’s correlation coefficients using the R package ‘corrplot’ (26); and (ii) Partial least squares discriminant analysis (PLS-DA) was performed using the R package ‘mixOmics’ (27) to evaluate significant starch parameters responsible for discriminating between waxy and non-waxy rice cultivars. A subset of the data was used for both statistical analyses. Rice cultivars with fewer than 3 records were excluded, so that a total of 12 Thai rice cultivars (KDML 105, Leuang 11, Leuang Pratew 123, Pathum Thani 1, Plai Ngahm Prachin Buri, Prachin Buri 1, RD10, RD6, San Pah Tawng, San Pah Tawng 1, Sao Hai and Suphan Buri 1) were used. In the present study, we considered only six starch parameters (percentage amylose, pasting temperature, peak viscosity, breakdown, final viscosity and setback), as they were the top most parameters reported in the curated studies. The dataset was log-2 transformed to conform more closely to normal distribution prior to calculations and the outputs have been presented as interactive graphs or tables on the website.

## Results and discussion

### Data characteristics

**Figure 1.**
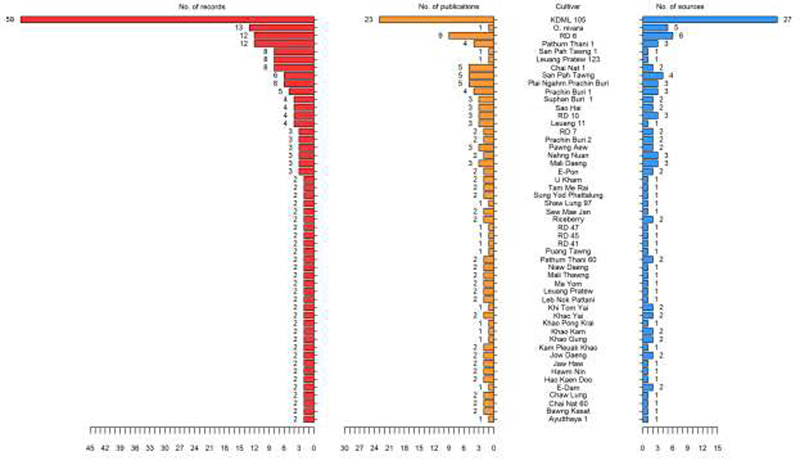
List of Thai rice cultivars in ThRSDB. The numbers of records (red), publications (yellow) and sources (blue) for each cultivar are illustrated. The cultivars with only one record are provided in Supplementary Table S1.

A wide variety of Thai rice cultivars from the published literature was collected in the database. All in all, there were 373 samples from 191 different Thai rice cultivars. The non-waxy and waxy rice cultivars with the greatest number of records and supporting studies were KDML 105 (the popular Thai jasmine rice) and RD6 (the famous Thai sticky rice), respectively (Figure 1 and Supplementary Table S1). KDML 105, *Oryza nivara* and RD6 were the top three rice cultivars supplied from several places (Figure 1). The KDML 105 samples from the research studies came from various areas of Thailand, except for the southern part, whereas the RD6 samples were majorly taken from the northeastern provinces (Supplementary Table S2). Of the 294 rice samples (approximately 80% of the total data), 254 were recorded as coming from 29 different provinces, 20 from 4 regions of Thailand and about 6% were commercially-branded products (Supplementary Table S2). Many of the rice cultivars (140 cultivars) have been understudied, with only one research study for each of these cultivars, mostly local landraces such as Ang Jerng Jahn, Bahng Gawk, Bal Cha Plau, to name a few (Supplementary Table S1).

The overall statistics of the different starch parameters are summarized in Table 1. In the current database, we collected 13 starch properties and 28 parameters. The percentage amylose and starch viscosity (except for peak temperature) were the parameters most frequently reported (i.e. more than 100 records). The percentage amylose is the key determinant of the cooked rice hardness, while the analysis of starch pasting behavior simulates food processing and can be used to correlate the starch functionality with its mechanical and structural properties (28, 29). On average, the percentage amylose and moisture content of the Thai rice cultivars were about 16.0% and 12.0%, respectively. The top two largest SEs in order were the SE of final and peak viscosity, while the amylopectin chain length distribution exhibited the lowest deviation. Similarly, the basic statistical description of the starch parameters of each cultivar is provided in Supplementary Table S3. Different numbers of starch properties were characterized for each cultivar. RD6, followed in order by KDML 105, Plai Ngahm Prachin Buri and Riceberry were the cultivars with the greatest number of starch properties reported. The highest percentage amylose was reported in Niaw Look Pueng and the lowest in San Pah Tawng 1. The final and peak viscosity values of Mali Daeng and Ayutthaya 1 were considerably high, with those of Leuang Pratew 123 and San Pah Tawng 1 were substantially lower.

**Table 1.** Summary of rice starch parameters in ThRSDB.

Property	Parameter	No. of records	Mean ± SE	Median	Min	Max
Moisture content	Moisture content (%)	160	12.23 ± 0.24	12.19	5.47	33.30
Amylose	Amylose (%)	325	16.05 ± 0.54	14.10	0	41.95
Amylose:amylopectin ratio	Amylose:amylopectin ratio	325	0.21 ± 0.01	0.16	0	0.72
Granule size	Mean diameter (µm)	32	52.17 ± 9.04	39.30	4.33	208.93
Crystallinity	Crystallinity (%)	36	22.20 ± 1.34	22.39	1.82	37.02
Amylopectin chain length distribution	Average chain length (glucose unit)	5	17.52 ± 0.22	17.30	17.10	18.29
	DP 6-12 (%)	22	33.79 ± 1.68	33.02	23.14	45.17
	DP 13-24 (%)	22	48.76 ± 1.09	48.22	35.50	56.73
	DP 25-36 (%)	22	7.15 ± 0.22	6.80	5.86	9.40
	DP ≥ 37 (%)	22	10.33 ± 1.55	10.76	1.21	24.02
Solubility	Solubility (%)	11	4.32 ± 0.74	4.70	1.14	7.80
Swelling power	Swelling power	12	27.93 ± 4.39	26.62	9.30	55.60
Gel consistency	Alkali	18	71.98 ± 3.66	75.67	38.50	98.10
	Neutral	2	86.35 ± 0.65	86.35	85.70	87.00
Gelatinization	T_o_ Gelatinization	59	66.70 ± 0.72	66.50	57.82	80.30
	T_p_ Gelatinization	59	72.47 ± 0.60	73.10	64.96	84.24
	T_c_ Gelatinization	59	79.67 ± 0.55	80.50	71.77	88.61
	ΔH Gelatinization	75	9.28 ± 0.48	9.90	0.70	17.04
	T_c_-T_o_ Gelatinization	26	10.63 ± 0.41	10.45	7.90	14.60
Retrogradation	Retrogradation (%)	9	47.34 ± 5.40	50.58	18.24	69.44
Viscosity	Pasting temperature	135	77.42 ± 0.71	76.64	56.20	95.55
	Peak viscosity	136	1073.06 ± 119.19	295.65	1.05	5277
	Peak temperature	2	68.75 ± 0.25	68.75	68.50	69
	Breakdown	126	442.61 ± 54.19	147.54	0.30	2540
	Final viscosity	130	1068.06 ± 127.80	319	0.53	5793
	Setback	136	406.38 ± 63.53	76	‒748	3078
Hydrolysis	Hydrolysis Index (HI)	3	64.77 ± 11.72	55.75	50.54	88.02
	Glycemic Index (GI)	3	71.66 ± 5.36	70.30	63.13	81.55

SE is standard error of the mean.

### Web interface

**Figure 2.**
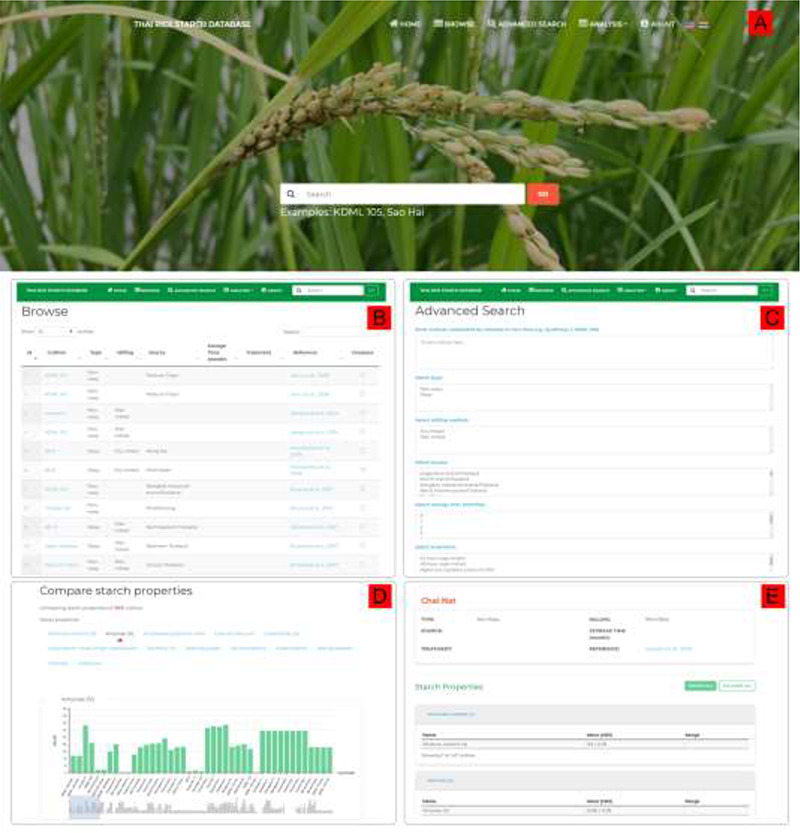
ThRSDB web interface. (A) Homepage with the top navigation menu and the Search form. The flag icons are for switching between English and Thai version. (B) Browse page. The Search box is provided on the top menu of every page except the homepage. (C) Advanced Search page. (D) A bar chart comparing the percentage amylose between cultivars. Other starch properties are listed, which can be selected to display the corresponding chart. (E) The page presenting detailed information on a selected cultivar.

A user-friendly web interface was implemented containing several functions for data access: Browse, Search and Advanced Search (Figure 2). The Browse function listed all cultivars in the database; Search allowed a query by the rice cultivar name; and Advanced Search enabled searching for more than one rice cultivar with various search options to constrain the query, such as waxy type, milling method, source, storage time and additional treatments. Each function was embedded into the top navigation menu. A search form was provided on the homepage. Within the Browse, Search Result and Advanced Search Result pages the users can render interactive bar charts comparing the starch properties of different rice cultivars (Figure 2D).

The detailed information on each cultivar was presented on a separate page (Figure 2E). This includes basic information on the rice cultivar as well as the key rice starch properties, composition, molecular structure and functionality, all acquired from the original study. The starch compositional parameters included moisture content, percentage amylose and calculated amylose:amylopectin ratio. The starch structural parameters were granule size, amylopectin chain length distribution and percent crystallinity, while the starch functional parameters covered gel consistency, gelatinization, retrogradation, viscosity, hydrolysis, swelling power and percent solubility. Users can access the cultivar information page by clicking on a rice cultivar listed on the Browse, Search Result and Advanced Search Result pages. ThRSDB is available in English and Thai language versions. The database is publicly available at http://thairicestarch.kku.ac.th.

### Analysis of correlation between starch parameters of Thai rice cultivars

The composition and molecular structure of starch are key determinants of its functional properties (2, 5, 28). The ability to infer the relationship between these compositional and functional properties using correlation analysis provides very useful knowledge for food product development. Therefore, it is important to test whether the large amount of data collected from various studies in ThRSDB could potentially be used to understand the relationship between the composition and molecular structure of starch and its functional properties.

**Figure 3.**
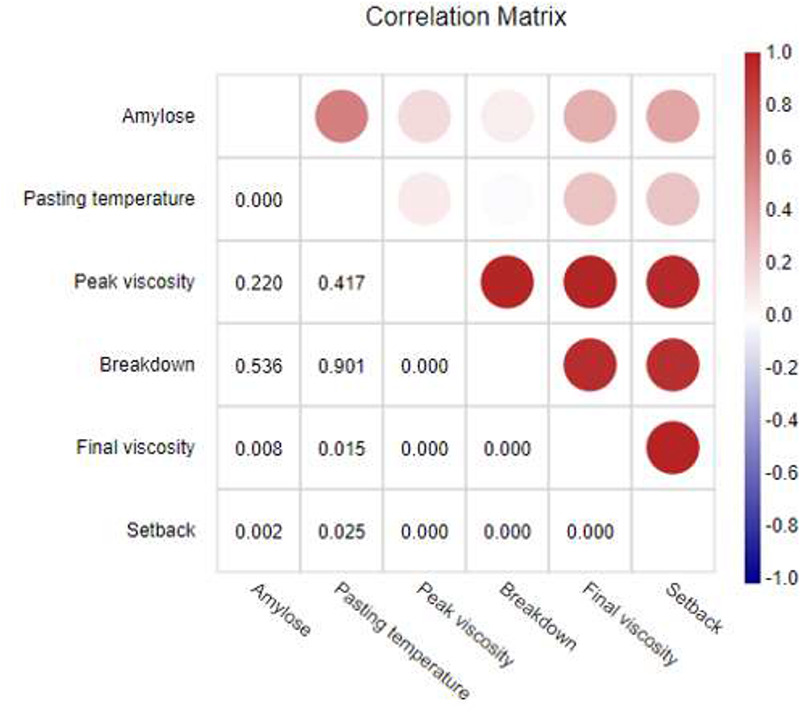
Correlation matrix between six starch parameters of Thai rice cultivars. The upper triangular matrix illustrates the correlation coefficients represented by a color on a continuous scale. The red color indicates a positive correlation, and blue color defines a negative correlation. The lower triangular matrix shows the *P*-values.

To achieve this aim, the parametric Pearson correlations between six starch parameters (including percentage of amylose, pasting temperature, peak viscosity, breakdown, final viscosity and setback) were calculated for a filtered set of the rice cultivars, which indicated the associations between the starch parameters (Figure 3). The correlations were considered as statistically significant at *P*-values of less than 0.05. It can be seen that the amylose content was significantly and positively correlated with the rheological properties, particularly the pasting temperature, final viscosity and starch setback. These relationships are consistent with those reported in previous studies on Thai rice cultivars, which showed that starch setback and pasting temperature increased with amylose concentration (7, 15, 30). Adding amylose to a rice starch paste can increase its storage modulus upon aging, which also supports the positive correlation reported between the percentage amylose and the final viscosity and setback (31). This example demonstrates that the wide range of rice starch data deposited in ThRSDB could be used for analyzing the correlation between starch structural content and its functional properties.

### Analysis of discrimination between waxy and non-waxy Thai rice cultivars

Rice starches from different cultivars possess distinct morphological, thermal and rheological characteristics (32, 33). These starch parameters can be used to discriminate different rice cultivars or different groups of cultivars. For example, Thai rice cultivars can be categorized by several criteria, based primarily on their starch properties such as amylose content, degree of gel consistency and waxiness (15, 34). The current database contains two main types of Thai rice cultivar: waxy and non-waxy rice. Both are valuable commodities in Thailand and in the worldwide economy.

**Figure 4.**
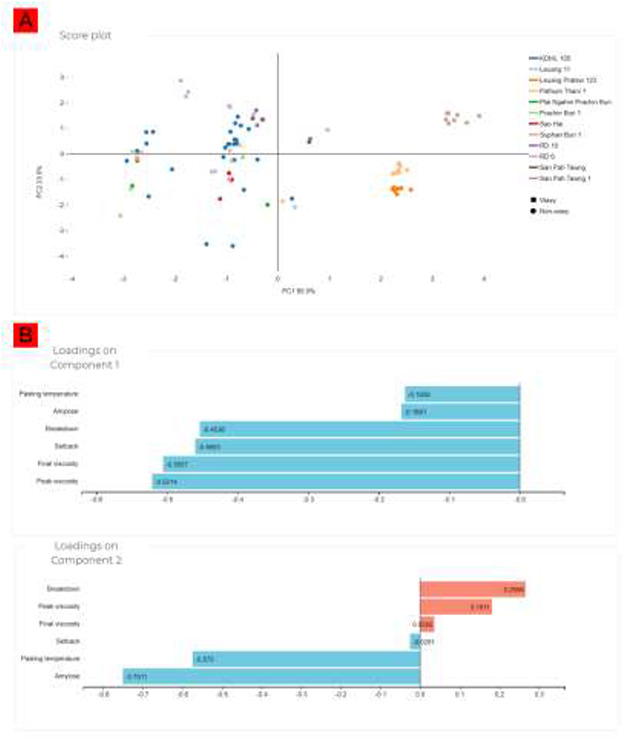
PLS-DA of the Thai rice cultivars. (A) Score plot showing the separation between the cultivars. (B) Loading plots indicating the influences of each starch parameter on the separation of the cultivars.

To demonstrate the utility of our curated data, we examined whether the Thai rice starch data collection in ThRSDB could correctly discriminate waxy from non-waxy rice. Therefore, PLS-DA was conducted to examine the variation among the Thai cultivars (Figure 4). PLS-DA is one of the dimensional reduction techniques (such as principal component analysis [PCA] and Orthogonal Projections to Latent Structures Discriminant Analysis [OPLS-DA]) that are widely used to explore variation in measured variables and to identify important variables for phenotypic discrimination (35). In this study, a multivariate classification model was built for the filtered starch data set, and the contribution of each starch parameter to the model was estimated as a loading score. The PLS-DA score plot displays the level of discrimination between the rice cultivars produced by the model (Figure 4A). It can be seen that a clear separation of the waxy rice cultivars (e.g. RD6, RD10, San Pah Tawng and San Pah Tawng 1) from the other non-waxy rice cultivars was achieved by the model. The loading plots show the magnitude of the separation and the direction of the differences for the starch parameters between the two cultivar types. The key discriminants for the two types of Thai rice cultivar were the variables, percentage amylose, pasting temperature and viscosity parameters (i.e. peak, final, breakdown and setback viscosity) (Figure 4B). This observation agrees with the characteristics of waxy and non-waxy rice which exhibit significant differences in amylose content and glutinous texture upon cooking (34). The waxy rice starch had a lower amylose content and displayed a greater swelling volume, which resulted in a higher viscosity than the non-waxy rice (15, 36). It is therefore feasible for researchers to utilize the ThRSDB data to categorize rice cultivars according to their starch characteristics.

## Conclusions and future directions

To the best of our knowledge, ThRSDB is the first platform where data on Thai rice starch composition, molecular structure and functionality from many sources of the scientific literature has been deposited and where this dispersed information has been made available in a well-organized and user-friendly version to the rice community. The database contains 28 essential parameters of 13 key starch properties, of which the parameters of percentage amylose, moisture content and starch viscosity were the most frequently studied. Of the two main types of cultivar, waxy and non-waxy rice, RD6 and KDML 105, respectively, were the most frequently reported.

We have demonstrated the value of the curated data through two successful analysis cases. Correlation analysis demonstrated that, with the extensively curated data, significant correlations could be observed between grain compositional parameters, such as percentage amylose and starch functionality parameters, such as pasting temperature and setback viscosity. PLS-DA also showed that the waxy and non-waxy rice cultivars could be separated based on the values of key discriminant parameters such as percentage amylose, pasting temperature and viscosity.

This digitalized, well-organized and searchable compendium of Thai rice starch data allows the rapid and efficient use of dispersed information on rice starch. It facilitates comparisons between rice cultivars and across research units. The database can be broadly applied in several aspects: for instance, it is possible to use the database in breeding programs through observing and comparing particular starch traits between two or more cultivars, through exploring the diversity of starch traits among different cultivars, or through preliminary screening for cultivars offering a specific starch trait. The broad continuum of industrial applications of starch also requires knowledge of its structural and functional properties, which can be easily obtained from ThRSDB. Besides, the source information shows the variety and availability of rice cultivars in different areas of Thailand. We believe that this information could be useful for research units, companies and other stakeholders and for economic planning. The raw data could be made available for download upon request, which allows extensive exploitation of the data with other information repositories.

As a starting point, ThRSDB currently focuses on starch information from Thai rice cultivars, an indica subspecies, under normal growing conditions. In the future, we plan to include other Asian rice cultivars such as japonica rice and long-grain basmati rice and to take account of variations in growing conditions so that the database will become a more comprehensive resource for rice starch information. As more research into rice starch properties is undertaken, we will continuously collect this new data and update the database. We are currently investigating to embed a data analysis module (e.g. correlation analysis), which would allow users to statistically analyze their own datasets. We believe that ThRSDB will be a valuable resource for the rice community worldwide and so will become a collaborative platform for sharing data in this research area.

## Limitations

A key limitation of ThRSDB is the incompleteness of the curated data. Most of the original research articles have only reported a limited number of grain quality traits because of differences in their research focus and technological concerns. Nevertheless, the grain amylose content was the most frequently characterized parameter. This parameter alone is of critical value to users because (i) it has long been proved to be an important trait for determining rice functionalities and grain qualities and (ii) it is widely used to categorize rice cultivars into different groups such as waxy, low amylose and high amylose rice.

Another limitation concerns the low amount of information on growth conditions. This is because the rice samples were mostly acquired from national rice research centers, private companies or markets. Experiments investigating a particular treatment such as elevated temperature, drought or soil salinity have been excluded from the current database.

## Supplementary Material

baaa068_Supp
