# Supplementary material for: ThRSDB: a database of Thai rice starch composition, molecular structure and functionality
Source: Database (Oxford). 2020 Dec 1;2020:baaa068. doi: 10.1093/database/baaa068 (PMC7706180; doi:10.1093/database/baaa068)
Supplement: baaa068_Supp [file baaa068_supp.zip › Supplementary TablesS1.docx]

**Supplementary Table S1.** List of Thai rice cultivars and the number of publications^1^.

| Cultivar | No. of Records | No. of Publications |
| --- | --- | --- |
| KDML 105 | 59 | 23 |
| O. nivara | 13 | 1 |
| Pathum Thani 1 | 12 | 4 |
| RD 6 | 12 | 9 |
| Chai Nat 1 | 8 | 5 |
| Leuang Pratew 123, San Pah Tawng 1 | 8 | 1 |
| Plai Ngahm Prachin Buri, San Pah Tawng | 6 | 5 |
| Prachin Buri 1 | 5 | 4 |
| Leuang 11, RD 10, Sao Hai, Suphan Buri 1 | 4 | 3 |
| Mali Daeng, Pawng Aew | 3 | 3 |
| E-Pon, Nahng Nuan, Prachin Buri 2, RD 7 | 3 | 2 |
| Bawng Kasat, Chai Nat 60, Chaw Lung, Hao Kaen Doo, Hawm Nin, Jaw Haw, Jow Daeng, Kam Pleuak Khao, Khao Yai, Leb Nok Pattani, Leuang Pratew, Ma Yom, Mali Thawng, Niaw Daeng, Pathum Thani 60, Riceberry, Sew Mae Jan, Sung Yod Phattalung, Tam Me Rai, U Kham | 2 | 2 |
| Ayutthaya 1, E-Dam, Khao Gung, Khao Kam, Khao Pong Krai, Khi Tom Yai, Puang Tawng, RD 41, RD 45, RD 47, Shaw Lung 97 | 2 | 1 |
| Ang Jerng Jahn, Bahng Gawk, Bal Cha Plau, Bal Khao Seu, Bal La Mi, Biaw Ku, Biaw Mai Yan Rai, Chai Nat, Chai Nat 2, Chao Daeng, Chao Yai, Chiang Phatthalung, Chiang rice, Daw Gaset, Daw Hahng Hee, Daw Nahng Nuan, Daw Nahng Nuan Lai, Daw Yuan, Dawk Kam, Dawk Pa-yawm, Dok Gian, E-Dahng, E-Khao, E-Khao Yai, E-Nawn, E-Non, E-Pae, E-Pua, E-Tia, Gaen Jan, Gai Ngaw, Gam, Gam Pun, Gra Dook Ngoo, Haek Yah, Hahng Yi 71, Hang Yee 71, Hantra 60, Hao Ma Phai, Hao Nah, Hawm, Hawm Janh, Hawm Pae, Hawm Sa-ngiam, Hom Viengping, Hua Nah, Jampah, Jaw Dam, Jaw Dam 25%, Jaw Dam 50%, Jaw Yai, Jek Chuey, Jow Daeng Daw, Jow Ne, Ka Saen, Kai Ngaw, Kam Nai, Kam Noi, Kam Poon, Kam Yai, KDML 105 mutant (HyKOS16, HyKOS21, HyKOS22, HyKOS3, HyKOS3-1, HyKOS7-1), Khai Mod Rin, Khao Bahn Nah 432, Khao Dam 50%, Khao Dam 90%, Khao Hawm, Khao Nak, Khao Ngan, Khao Pitsanulok, Khao Rai Kaset, Khao Ruang Yao, Khaw Kaw, Khaw Niew Dam, Khaw-Tahang, Kheaw-Prachin Buri, Khem Tawng, Khi Tom, Khi Tom Hahng Nahk, Khiaw Ngoo, Kordeaw, Lam Tan, Lao Taek, Leuang, Leuang 152, Leuang Awn, Leuang Bun Mah, Leuang Lao Khan, Mae Hahng, Mali (breeding line 10), Mali Dam, Mali Hawm, Muay Hin, Nahm Man Nghua, Nahng Hok, Nahng Kong, Nahng Ni, Nahng Payah 132, Nam Ang, Nauykaur, Ngan Khao, Niang Mow, Niaw Dam Pleuak Dam, Niaw Dam Pleuak Khao, Niaw Dawk Yong, Niaw Lan Tan, Niaw Look Pueng, Pa Sew, Pa-mah, Peuang Nam, Phatthalung 60, Phitsanulok, Phitsanulok 3, Phitsanulok 60-1, Phitsanulok 60-2, Pla Khaeng, Pla Sew, Pratum, Puang Rai 2, RD 13, RD 15, RD 27, Saen Sa-bai, Sakon Nakhon, San Huang Khao, San Pah Tawng Daw, San Plah Laht, Sang Yod, Soh Mah Lee, Srirak, Suphan Buri 90, Traditional waxy rice, Wild rice, Yeun Kalasin, Yi Pun, Yim | 1 | 1 |

^1^ The table shows the number of records and the number of published articles of each Thai rice cultivar.
