# Supplementary material for: ThRSDB: a database of Thai rice starch composition, molecular structure and functionality
Source: Database (Oxford). 2020 Dec 1;2020:baaa068. doi: 10.1093/database/baaa068 (PMC7706180; doi:10.1093/database/baaa068)
Supplement: baaa068_Supp [file baaa068_supp.zip › Supplementary TablesS2.docx]

**Supplementary Table S2.** List of sources and supplied cultivars^2^.

| Source | No. of records | No. of cultivars | List of cultivars |
| --- | --- | --- | --- |
| By province | | | |
| Phatthalung | 28 | 25 | Bahng Gawk, Chaw Lung, Chiang Phatthalung, Chiang rice, Gaen Jan, Haek Yah, Hawm Janh, Jampah, Khai Mod Rin, Khao Ruang Yao, Khem Tawng, Leuang 152, Nahng Payah 132, Nauykaur, Niaw Dam Pleuak Dam, Niaw Dam Pleuak Khao, Niaw Dawk Yong, Niaw Lan Tan, Niaw Look Pueng, Peuang Nam, Phatthalung 60, Puang Tawng, Sang Yod, Srirak, Sung Yod Phattalung |
| Mukdahan | 27 | 19 | Daw Gaset, Daw Yuan, E-Pae, E-Pon, E-Pua, Gam, Gam Pun, Hahng Yi 71, Hao Kaen Doo, Hao Ma Phai, Ka Saen, Kam Poon, Khao Kam, Khao Rai Kaset, Ma Yom, O. nivara, Pawng Aew, San Huang Khao, U Kham |
| Kalasin | 22 | 19 | Daw Nahng Nuan, Dok Gian, E-Dam, E-Khao, E-Pon, Gra Dook Ngoo, Kam Nai, Khao Gung, Khao Ngan, Khao Yai, Khi Tom Yai, Mae Hahng, Mali Daeng, Nahng Nuan, Ngan Khao, O. nivara, Pla Sew, San Pah Tawng, Yi Pun |
| Pathum Thani | 18 | 8 | Chai Nat 60, KDML 105, Mali Thawng, Pathum Thani 60, RD 13, RD 15, RD 27, Suphan Buri 1 |
| Chiang Mai | 18 | 10 | Hom Viengping, KDML 105, KDML 105 mutant (HyKOS16), KDML 105 mutant (HyKOS21), KDML 105 mutant (HyKOS22), KDML 105 mutant (HyKOS3-1), KDML 105 mutant (HyKOS3), KDML 105 mutant (HyKOS7-1), Khao Pong Krai, San Pah Tawng 1 |
| Sakon Nakhon | 14 | 13 | Daw Nahng Nuan Lai, E-Dam, E-Khao Yai, E-Tia, Gai Ngaw, Kai Ngaw, Khi Tom Hahng Nahk, Khi Tom Yai, Nahm Man Nghua, Nahng Ni, Nam Ang, O. nivara, Pa Sew |
| Ratchaburi | 12 | 2 | Chai Nat 1, Leuang Pratew 123 |
| Surin | 11 | 7 | Bawng Kasat, Chao Daeng, Niang Mow, RD 10, RD 6, San Pah Tawng, Wild rice |
| Prachinburi | 11 | 5 | Ayutthaya 1, Plai Ngahm Prachin Buri, Prachin Buri 1, Prachin Buri 2, RD 45 |
| Maha Sarakham | 10 | 8 | KDML 105, Khao Gung, Khi Tom, Leuang Bun Mah, Nahng Nuan, O. nivara, RD 6, San Pah Tawng Daw |
| Chiang Rai | 10 | 9 | Bal Cha Plau, Bal Khao Seu, Bal La Mi, Biaw Ku, Biaw Mai Yan Rai, Jaw Haw, Jow Ne, Phitsanulok, Yim |
| Suphan Buri | 8 | 8 | Jaw Dam, Jaw Dam 25%, Jaw Dam 50%, Khao Dam 50%, Khao Dam 90%, Khao Hawm, Mali (breeding line 10), Mali Hawm |
| Chachoengsao | 8 | 1 | Pathum Thani 1 |
| Phitsanulok | 7 | 5 | Khao Pitsanulok, Phitsanulok 60-1, Phitsanulok 60-2, RD 41, RD 47 |
| Khon Kaen | 7 | 2 | KDML 105, RD 6 |
| Nakhon Ratchasima | 6 | 6 | Chao Yai, Jaw Yai, KDML 105, Khaw Niew Dam, Pratum, RD 6 |
| Yasothon | 5 | 4 | E-Nawn, E-Non, Hawm Pae, KDML 105 |
| Phetchaburi | 5 | 4 | Ang Jerng Jahn, Jek Chuey, Leuang Pratew, Puang Rai 2 |
| Songkhla | 4 | 3 | Hao Nah, Hua Nah, Tam Me Rai |
| Saraburi | 4 | 2 | Khaw Kaw, Sao Hai |
| Roi Et | 4 | 3 | Jow Daeng, KDML 105, Khao Kam |
| Pattani | 4 | 2 | Leb Nok Pattani, Shaw Lung 97 |
| Nakhon Phanom | 4 | 2 | KDML 105, O. nivara |
| Ubon Ratchathani | 2 | 1 | KDML 105 |
| Phetchabun | 1 | 1 | Riceberry |
| Nong Kai | 1 | 1 | RD 6 |
| Mae Hong Son | 1 | 1 | San Pah Tawng |
| Kanchanaburi | 1 | 1 | Leuang Lao Khan |
| Buri Ram | 1 | 1 | Khao Nak |
| By region | | | |
| Northeastern Thailand | 11 | 3 | KDML 105, RD 10, RD 7 |
| Central Thailand | 6 | 6 | Chai Nat 2, Pathum Thani 1, Pathum Thani 60, Plai Ngahm Prachin Buri, Prachin Buri 1, Prachin Buri 2 |
| Northern Thailand | 2 | 2 | Phitsanulok 3, Sakon Nakhon |
| Southern Thailand | 1 | 1 | Dawk Pa-yawm |
| Others | | | |
| Thai-branded products (12) | 13 | 1 | KDML 105 |
| US-branded products (3) | 3 | 1 | KDML 105 |
| Thai-branded product (1) | 2 | 2 | KDML 105, Mali Daeng |
| Laos | 2 | 2 | Dawk Kam, Traditional waxy rice |

^2^The table shows the number of records and the list of cultivars collected from each source. Sources are grouped by province, region and other for visualization purpose. Number of individual brands are in parentheses.
